# The complete chloroplast genome sequence of *Tribulus terrestris*, an important traditional Chinese medicine

**DOI:** 10.1080/23802359.2019.1667891

**Published:** 2019-09-20

**Authors:** Jiakun Yan, Ningning Zhang, Yizhong Duan

**Affiliations:** Shaanxi Key Laboratory of Ecological Restoration in Shanbei Mining Area, Yulin University, Yulin, Shaanxi, China

**Keywords:** Tribulus terrestris, chloroplast genome, phylogenetic analysis

## Abstract

*Tribulus terrestris* is an important traditional medicine in China, which is widely distributed in north China. Here, the chloroplast genome sequences were detected. The chloroplast genome of *T. terrestris* is circular-mapping molecule of 158,184 bp in size, which consisted of a pair of inverted repeat regions of 25,842 bp each, a large single copy region of 88,878 bp, and a small single copy region of 17,622 bp. A total of 129 genes were annotated, including 37 tRNA, 8 rRNA, and 84 protein-coding genes. Phylogenetic analysis showed *T. terrestris* clustered with Krameria lanceolate and Krameria bicolor.

*Tribulus terrestris*, an important traditional medicine in China, is widely distributed in north China. In clinical practice, the dry fruit of *T. terrestris* is used as one of the ingredients of traditional Chinese medicine compound to cure the headache diziness, chest coerces bloated pain and red eyes (Ren et al. 2019). In modern medicine, *T. terrestris* and the products of *T. terrestris* are found to function in many diseases because it contains active constituents for therapeutic values (Ghazala et al. 2019). In addition, *T. terrestris* also plays important role in control of desertification, especially in arid and semi-arid region like the Northwest China (Chen et al. 2014). Taking all these into conclusion, we could find that the *T. terrestris* is one of important plant species, which has double function in medicaments and ecology. However, at present, the research about plant biology of *T. terrestris* was little. There is no complete chloroplast (cp) genome sequence of *T. terrestris* in the GenBank database. To provide a better understanding on the evolution and genetics of *T. terrestris* and other species, we assemble and characterize *T. terrestris*’cp genome.

The chloroplast genome DNA was extracted from fresh leaves collected from a naturally grown plant in Mu Us Sandland, Yulin, Shaan Xi province, China. The voucher specimen (20190525YL02) was deposited in the herbarium of Yulin University. Total 16,744,698 paired-end reads of 150 bp readers were obtained by sequencing using an Illumina HiSeq X Ten platform and 208,789 reads were used to assemble the cp genome. The assembled cp genome (GenBank accession MN164624) was annotated using the online annotation tool DOGMA (Wyman et al. 2004) and further corrected manually (Yan et al. 2013).

The chloroplast genome of *T. terrestris* is circular-mapping molecule of 158,184 bp in size, which consisted of a pair of inverted repeat regions of 25,842 bp each, a large single copy region of 88,878 bp, and a small single copy region of 17,622 bp. A total of 129 genes were annotated, including 37 tRNA, 8 rRNA, and 84 protein-coding genes.

A phylogenetic analysis was carried out with *T. terrestris* and seven other complete cp genomes collected from Genbank including *Nicotiana tabacum* (Z00044.2), *Pithecellobium flexicaule* (KX852444), *Larrea tridentata* (MK726018), *Krameria lanceolata* (MK726016), *Krameria bicolor* (MK726015), *Guaiacum angustifolium* (MK726011), *Tetraena mongolica* (MH325021) using clustalX (Larkin et al. 2007). The results showed that *T. terrestris* clustered with *Krameria lanceolate* and *Krameria bicolor* (Figure 1).

**Figure 1.**
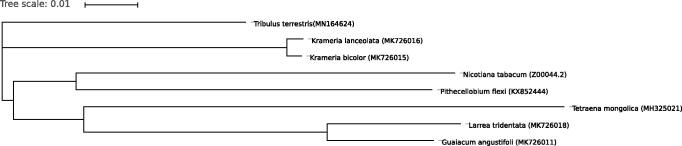
Phylogenetic tree based on 8 complete chloroplast genome sequences.
